# Immunoglobulin superfamily 6 is a molecule involved in the anti-tumor activity of macrophages in lung adenocarcinoma

**DOI:** 10.1186/s12885-023-11681-w

**Published:** 2023-11-30

**Authors:** Qisi Zheng, Ting Wang, Gechen Jiang, Miao Li, Zhi Zhang, Yuxin Chen, Xinyu Tian

**Affiliations:** 1 Department of Laboratory Medicine, Nanjing Drum Tower Hospital Clinical College of Nanjing Medical University, Nanjing, China; 2 Department of Laboratory Medicine, Nanjing Drum Tower Hospital, Nanjing University Medical School, Nanjing, China; 3 Department of Laboratory Medicine, Jiangsu Cancer Hospital & Jiangsu Institute of Cancer Research & The Affiliated Cancer Hospital of Nanjing Medical University, Nanjing, China; 4 Department of Thoracic Surgery, Jiangsu Cancer Hospital & Jiangsu Institute of Cancer Research & The Affiliated Cancer Hospital of Nanjing Medical University, Nanjing, China

**Keywords:** Immunoglobulin superfamily 6, Lung adenocarcinoma, Macrophages, Anti-tumor activity

## Abstract

**Background:**

Immunoglobulin superfamily 6 (IGSF6) is a novel member of the immunoglobulin superfamily and has been implicated in various diseases. However, the specific role of IGSF6 in the anti-tumor immunity within lung adenocarcinoma (LUAD) remains unclear.

**Methods:**

We analyzed the *IGSF6* expression in LUAD using data from TCGA, and we performed qRT-PCR and western blotting to validate these findings using tissue samples obtained from LUAD patients. Images of IHC staining were obtained from HPA. To assess the clinical relevance of *IGSF6* expression, we utilized UALCAN and SPSS to analyze its association with major clinical features of LUAD. Additionally, we employed ROC curves and survival analysis to evaluate the potential diagnostic and prognostic value of *IGSF6* in LUAD. To gain insights into the functional implications of IGSF6, we performed enrichment analysis using the R software *clusterProfiler* package. Moreover, we utilized TIMER2.0 and TISIDB to investigate the relationship between *IGSF6* and immune infiltrates in LUAD. The proportion of tumor-infiltrating immune cells in LUAD was assessed using FCM, and their correlation with *IGSF6* expression in tumor tissues was analyzed. The localization of IGSF6 protein on macrophages was confirmed using the HPA and FCM. To determine the regulatory role of IGSF6 on macrophage activity in LUAD, we employed ELISA, FCM, and tumor-bearing models.

**Results:**

We discovered that both IGSF6 mRNA and protein levels were significantly decreased in LUAD. Additionally, we observed a negative correlation between *IGSF6* expression and TNM stages as well as pathologic stages in LUAD. Notably, *IGSF6* exhibited high sensitivity and specificity in diagnosing LUAD, and was positively associated with the survival rate of LUAD patients. Furthermore, *IGSF6* expression was closely linked to gene sets involved in immune response. *IGSF6* expression showed a positive correlation with immune infiltrates exhibiting anti-tumor activity, particularly M1 macrophages. We confirmed the predominant localization of the IGSF6 protein on the membrane of M1 macrophages. Importantly, the knockdown of IGSF6 resulted in a reduction in the anti-tumor activity of M1 macrophages, thereby promoting tumor progression.

**Conclusion:**

IGSF6 is a molecule that is essential for the anti-tumor activity of macrophages in LUAD.

**Supplementary Information:**

The online version contains supplementary material available at 10.1186/s12885-023-11681-w.

## Introduction

Lung cancer is a prevalent and highly fatal cancer, causing millions of deaths worldwide each year. Unfortunately, many lung cancer patients are diagnosed at advanced stages, and treatment options with curative potential are often limited [1, 2]. Among the different subtypes of lung cancer, lung adenocarcinoma (LUAD) is the most common, accounting for 50% of all lung cancer cases, and its incidence continues to rise [3]. Immunotherapy has emerged as a promising approach for the treatment of various cancers, including LUAD. However, the effectiveness of immunotherapy in LUAD is often hindered by immune suppression [4, 5]. Therefore, there is a critical need to reverse the immunosuppression in LUAD to enhance the efficiency of immunotherapy.

Macrophages are immune cells that play a crucial role in the immune response and maintaining tissue balance [6]. In the tumor microenvironment (TME), macrophages can polarize into two subtypes: M1 and M2 macrophages [7]. M1 macrophages exhibit anti-tumor activity and primarily inhibit tumor growth and metastasis by producing inflammatory mediators and cytokines, such as tumor necrosis factor-alpha (TNF-α), interleukin-12 (IL-12), and interleukin-6 (IL-6). These molecules activate immune cells and induce tumor cell apoptosis. Additionally, M1 macrophages promote T-cell activation and proliferation by expressing antigen-presenting molecules and co-stimulatory molecules, thereby enhancing the anti-tumor immune response [8–11]. On the contrary, M2 macrophages possess immunosuppressive and tumor-promoting characteristics. They primarily suppress immune cell activation and promote tumor cell growth and invasion by secreting anti-inflammatory cytokines, such as interleukin-10 (IL-10) and transforming growth factor-beta (TGF-β). Furthermore, M2 macrophages contribute to tumor angiogenesis and tissue remodeling, providing support for tumor growth and metastasis [12–14]. In LUAD, the abundance of M2 macrophages is often higher than M1 and associated with a higher malignancy degree and worse prognosis. The presence of M2 macrophages inhibits anti-tumor immunity and tumor cell apoptosis, thereby promoting tumor progression [14–16]. Therefore, balancing the polarization and function of macrophages may be an important strategy for LUAD treatment, which can be achieved by inhibiting the activation of M2 macrophages or promoting the activation of M1 macrophages [17, 18].

Immunoglobulin superfamily 6 (IGSF6), also known as DOwn-Regulated by Activation (DORA), is a novel member of the immunoglobulin superfamily. It is encoded by the IGSF6 gene located at 16p11-p12 in humans [19, 20]. IGSF6 is highly expressed in the spleen, lymph nodes, and peripheral blood lymphocytes, but low in other tissues like bone marrow, thymus, fetal liver, and appendix [21, 22]. The IGSF6 protein is identified as a receptor of the CD8 family, which contains a single V-type loop domain with an associated J chain region, a transmembrane region with an atypical tyrosine residue, and a cytoplasmic domain with three putative tyrosine phosphorylation sites. Current studies suggest that IGSF6 is restricted to the myeloid lineage and may function as a co-receptor in the antigen uptake complex of dendritic cells (DCs) [19]. Additionally, IGSF6 has been associated with various diseases such as inflammatory bowel disease, atherosclerosis, and Parkinson’s disease [23–26]. However, its role in the anti-tumor immune response within LUAD is still not well understood.

In this study, we identified the levels of IGSF6 in LUAD and analyzed its role in the diagnosis and prognosis of LUAD patients. Furthermore, we examined the gene sets associated with *IGSF6* in LUAD and explored the correlation between *IGSF6* expression and immune cell infiltration in LUAD. Lastly, we discovered the crucial role of IGSF6 in the anti-tumor activity of M1 macrophages in LUAD.

## Methods

### Data collection and analysis

RNAseq data was downloaded and curated from the TCGA-LUAD project using the STAR pipeline from the TCGA database (https://portal.gdc.cancer.gov). The data was extracted in TPM format without any filtering. We processed the data using log2(value + 1) transformation. To ensure appropriate statistical analysis, we utilized suitable statistical methods using the *stats* package and *car* package. If the data did not meet the requirements for statistical analysis, it was excluded from further analysis. Tumor immune estimation resource, version 2 (TIMER2.0) (http://timer.comp-genomics.org/timer/) was used to analyze the expression of *IGSF6* in different cancers based on the data obtained from TCGA. Additionally, to confirm *IGSF6* expression in LUAD, data from the TCGA database were analyzed using the gene expression profiling interactive analysis 2 (GEPIA2) (http://gepia2.cancer-pku.cn/#index).

### Immunohistochemistry (IHC) staining

IHC images of IGSF6 protein levels in normal tissues and LUAD tissues were downloaded from the HPA (http://www.proteinatlas.org/).

### Patients and samples

In this study, tumor tissues and adjacent normal tissues were collected from LUAD patients who underwent primary surgical resection. A total of 93 tumor tissue samples and 93 adjacent normal tissue samples were included in the analysis, with 55 samples being matched. The present study was approved by the Ethics Committee of The Affiliated Cancer Hospital of Nanjing Medical University and the Ethics Committee of Nanjing Drum Tower Hospital (No. 2021-651-02). Written informed consent was obtained from all the subjects following the Declaration of Helsinki. The clinicopathological characteristics of the study cohort are presented in Table 1.

**Table 1:** Correlation between *IGSF6* expression and clinicopathological characteristics of LUAD patients (*)

Characteristics	Total(N)	IGSF6 relative expression	
		Mean (95% CI)	*p* value
**Age**	93		
<=65	55	0.9856 (0.8309-1.1400)	Reference
> 65	38	1.2490 (0.9825–1.5150)	0.0738
**Gender**	93		
Female	48	1.037 (0.8623–1.2110)	Reference
Male	45	1.165 (0.9284–1.4030)	0.3817
**IGSF6**	93		
Low	48	0.4620 (0.3978–0.5263)	Reference
High	45	1.6900 (1.5540–1.8260)	**<0.001**
**T stage**	93		
T1	55	1.4630 (1.2490–1.6770)	Reference
T2	26	1.0110 (0.8476–1.1740)	**0.0085**
T3	9	0.4814 (0.4119–0.5510)	**< 0.001**
T4	3	0.1819 (0.04829–0.3155)	**< 0.001**
**M stage**	93		
M0	89	1.1400 (0.9737–1.3060)	Reference
M1	4	0.9159 (0.6323-1.2000)	0.2480
** N stage**	93		
N0	63	1.693 (1.522–1.863)	Reference
N1	21	1.058 (0.7900–1.325)	**< 0.001**
N2	6	0.4223 (0.3385–0.5060)	**< 0.001**
N3	3	0.4736 (0.3013–0.6460)	**< 0.001**
**Pathologic stage**	93		
Stage I	59	1.676 (1.532–1.820)	Reference
Stage II	21	0.6413 (0.5302–0.7523)	**< 0.001**
Stage III	10	0.3295 (0.2255–0.4335)	**< 0.001**
Stage IV	3	0.0802 (0.0675–0.0929)	**< 0.001**

(*): The 2^−ΔCT^ calculation method; N: number

### Western blotting

Protein extraction from cells or tissues was prepared as described previously [27]. Protein (30 µg) was separated by 12% SDS-PAGE, and the gels were cut according to the marker indications and the position of the target bands. Protein was then transferred onto Immobilon polyvinylidene fluoride membranes (BioRad, Hercules, CA) that had been cut to the size of the gels. The membranes were blocked with 30 mL 5% BSA before probing overnight at 4℃ with rabbit-anti-human IGSF6 polyclonal antibody (1:1000) (ThermoFisher Scientific, Dallas, TX) or rabbit-anti-human β-ACTIN antibody (1:1000) (CST, Danvers, MA). After primary antibody incubation, the membranes were washed and incubated with a secondary HRP-conjugated goat anti-rabbit IgG (diluted at 1:8000) (CST, Danvers, MA), followed by chemiluminescent detection (Champion Chemical, Whittier, CA). Full-length blots were included in the supplementary material (Additional file 1).

### RNA isolation and qRT-PCR

Total RNA was isolated from tissues or cells with TRIzol reagent (Invitrogen, Carlsbad, CA) according to the manufacturer’s instructions [28]. The isolated RNA was then reverse-transcribed into cDNA using the HiScript 1st Strand cDNA Synthesis Kit (Vazyme, Nanjing, JS). Quantitative PCR was conducted using AceQ qPCR SYBR Green Master Mix (Vazyme, Nanjing, JS) on a Bio-Rad CFX96 Real-Time PCR system (BioRad, Hercules, CA). The thermal cycling conditions consisted of an initial denaturation at 95 ℃ for 5 min, followed by 40 cycles of amplification at 95 ℃ for 10 s, 60 ℃ for 30 s and 72℃ for 30s. The primer sequences used were as follows: human β-ACTIN, sense 5-GAGTGTGGAGACCATCAAGGA-3, antisense 5-TGTATTGCTTTGCGTTGGAC-3; human IGSF6, sense 5-GCAATCTCGGCTCACTACAACCTC-3, antisense 5-CGTGGTGGTGCGTACCTGTAATC-3. The representative amplification plot and melt curve were provided in the supplementary material (Additional file 2). Each sample had three replicate wells and three independent experiments were performed. The relative levels of the target gene were determined using the 2^−ΔΔCT^ calculation method.

### UALCAN analysis

The UALCAN (http://ualcan.path.uab.edu/) website provides a comprehensive and interactive analysis of bioinformatics using RNA-seq and clinical data from 33 types of cancer in the TCGA. This website enables users to compare gene expression and promoter methylation levels between tumors and healthy samples, as well as analyze their relationship with different stages or subtypes of tumors and other clinicopathological features. In the present study, the association of *IGSF6* expression with clinicopathological parameters of LUAD was analyzed using UALCAN. Furthermore, promoter methylation levels of *IGSF6* in normal tissues and primary tumors were also estimated using UALCAN.

### Survival prognosis analysis

Kaplan-Meier survival curves were used to evaluate the association of overall survival (OS), disease special survival (DSS), and progress-free interval (PFI) with *IGSF6* expression in LUAD. The expression levels were divided into high-expression and low-expression groups using a cutoff value of 50%. The hazard ratio (HR) with 95% confidence intervals was also analyzed, as well as the log-rank *p*-value. Statistical significance was defined as *p* < 0.05.

### *IGSF6*-related gene enrichment analysis

Pearson correlation analysis was performed to investigate the correlation between *IGSF6* and other genes in LUAD using TCGA data. The top 300 genes that exhibited the strongest correlation with *IGSF6* were selected for enrichment analysis. Gene Ontology (GO) and Kyoto Encyclopedia of Genes and Genomes (KEGG) analyses were conducted by running the *clusterProfiler* package in R software. To identify different gene expression profiles between the high- and low-risk subgroups, genes with a fold change greater than |log2FC|>1 and a false discovery rate (FDR) less than 0.05 were considered significant. The Benjamini-Hochberg (BH) method was used to adjust the *p*-value. Detailed parameters were shown as the following: ont = all, *q*-value-Cutoff = 0.05, and *p*-value-Cutoff = 0.05. Moreover, gene set enrichment analysis (GSEA) was conducted using the gseKEGG and gsePathway functions in *clusterProfiler*. The parameters for GSEA included maxGSSize = 1000, minGSSize = 10, nPerm = 1000, and *p*-value-Cutoff = 0.05.

### Immune cell infiltration

TIMER2.0 and Tumor Immune System Interaction Database (TISIDB) (http://cis.hku.hk/TISIDB/index.php) were utilized to investigate the correlation between *IGSF6* and tumor-infiltrating immune cells in LUAD.

### Prediction of IGSF6 localization in cells

Enrichment of IGSF6 in different cell populations within lung tissues and predictions of IGSF6 localization in cells were obtained from the HPA database.

### Flow cytometry (FCM)

Tumor tissues from LUAD patients were cut into small pieces (1–2 mm^3^) and digested with collagenase II (Sigma-Aldrich, St. Louis, MO) at 37 °C for 2 h on a rotating platform to obtain single-cell suspensions. Mononuclear cells were isolated by density gradient centrifugation over a Percoll cushion (Sigma-Aldrich, St. Louis, MO). The cells were collected and stained with PE-anti-human-CD86 (5µL/1 × 10^6^ cells/100µL) (eBioscience, San Diego, CA) and PE/CY5-anti-human-CD11b (5µL/1 × 10^6^ cells/100µL) (Biolegend, San Diego, CA) for M1 macrophages, APC-anti-human-CD206 (5µL/1 × 10^6^ cells/100µL) (eBioscience, San Diego, CA) and PE/CY5-anti-human-CD11b (5µL/1 × 10^6^ cells/100µL) (Biolegend, San Diego, CA) for M2 macrophages, and PE-anti-human-CD123 (5µL/1 × 10^6^ cells/100µL) (eBioscience, San Diego, CA) and FITC-anti-human-CD11c (eBioscience, San Diego, CA) (5µL/1 × 10^6^ cells/100µL) for immature DCs (iDCs).

To detect the proportion of activated Th1, mononuclear cells (1 × 10^6^ cells) from tumor tissues were stimulated with 50 ng/mL of PMA (Sigma-Aldrich, St. Louis, MO) and 1 µg/mL of ionomycin (Sigma-Aldrich, St. Louis, MO) for 2 h and then incubated for an additional 4 h in the presence of 1 µg/mL of brefeldin-A (eBioscience, San Diego, CA). The cells were then stained with FITC-anti-human-CD4 (5µL/1 × 10^6^ cells/100µL) (eBioscience, San Diego, CA), fixed, permeabilized, and stained with PE-anti-human-IFN-γ (5µL/1 × 10^6^ cells/100µL) (eBioscience, San Diego, CA) according to the Intracellular Staining Kit (Invitrogen, Carlsbad, CA) instructions.

To confirm the localization of IGSF6 on M1 macrophages, mononuclear cells from tumor tissues were stained with FITC-anti-human-IGSF6 (5µL/1 × 10^6^ cells/100µL) (Santa Cruz, Santa Cruz, CA), PE-anti-human-CD86 (5µL/1 × 10^6^ cells/100µL) (eBioscience, San Diego, CA) and PE/CY5-anti-human-CD11b (5µL/1 × 10^6^ cells/100µL) (Biolegend, San Diego, CA) for 30 min and then were analyzed by flow cytometer (FACSAria II, BD, NJ). Additionally, induced M1 macrophages were stained with PE-anti-human-CD86 (5µL/1 × 10^6^ cells/100µL) (eBioscience, San Diego, CA) and FITC-anti-human-IGSF6 (5µL/1 × 10^6^ cells/100µL) (Santa Cruz, Santa Cruz, CA) for 30 min and then were analyzed by flow cytometer (FACSAria II, BD, NJ).

### Cell culture

A549 cells and human monocyte/macrophage cell line THP-1 were purchased from the Cell Bank of the Chinese Academy of Sciences (Shanghai, China). THP-1 cells were cultured in RPMI-1640 (Gibco, Carlsbad, MA) with 10% FBS (Gibco, Carlsbad, MA) at 5% CO_2_, 37℃. THP-1 cells are suspension cells that thrive in a slightly acidic environment. Therefore, when the medium turns slightly yellow (orange-red), it indicates the need for either replenishing or partially changing the medium. It is important to maintain the culture density between 4 ~ 8 × 10^5^/mL, and if it exceeds 2 × 10^6^/mL, passage is necessary. THP-1 cells are particularly sensitive to mechanical forces and should be handled with care to minimize any disturbances during regular culture. A549 cells were cultured in DMEM (Gibco, Carlsbad, MA) with 10% FBS (Gibco, Carlsbad, MA) at 5% CO_2_, 37℃.

### Induction of M1 macrophages

THP-1 cells (1 × 10^6^ cells/mL) cultured in RPMI 1640 medium (Gibco, Carlsbad, MA) were stimulated with PMA (100 ng/mL) (Sigma-Aldrich, California, CA) for 24 h. After that, the cells were co-cultured with 20ng/mL IFN-γ, 100ng/mL LPS, and 50ng/mL GM-CSF (Peprotech, Rocky Hill, NJ) for 48 h to generate M1 macrophages.

### siRNA assay

IGSF6 siRNA (50 nM) or its negative control (50 nM) (Ribobio Co., Guangzhou, GD) was transfected into THP-1-induced M0 macrophages plated in 48-well plates with Lipofectamine 2000 (Invitrogen, Carlsbad, CA) according to the manufacturer’s protocol. The transfected cells were then co-cultured with 20ng/mL IFN-γ, 100ng/mL LPS, and 50ng/mL GM-CSF (Peprotech, NJ, USA) for 48 h. To detect the levels of CD86 and HLA-DR on the cell surface, cells were stained with PE-anti-human-CD86 (5µL/1 × 10^6^ cells/100µL) (eBioscience, San Diego, CA) and APC-anti-human-HLA-DR (5µL/1 × 10^6^ cells/100µL) (eBioscience, San Diego, CA) for 30 min and then analyzed by flow cytometer (FACSAria II, BD, NJ). The production of IL-12 and TNFα was detected with the specific ELISA kits (Invitrogen, Carlsbad, CA) and quantified following the manufacturer’s instructions.

### In vivo experiments

To investigate the influence of IGSF6 on the anti-tumor activity of M1 macrophages in vivo, A549 cells (1 × 10^6^ cells/mouse) and M1 with IGSF6 knockdown (2 × 10^6^ cells/mouse) were injected subcutaneously into nude mice, and the tumor progression was continuously monitored. In another experiment, A549 cells (1 × 10^6^ cells/mouse), M1 with IGSF6 knockdown (2 × 10^6^ cells/mouse), and CD4^+^ T cells from PBMCs of healthy donors (2 × 10^6^ cells/mouse) were injected subcutaneously into nude mice, and the tumor progression was continuously monitored. All of the experiments were approved by the Animal Use Committee of Nanjing Medical University following the International Guiding Principles for Biomedical Research Involving Animals.

### Statistical analysis

Statistical analysis was performed with SPSS 21.0 (IBM, Chicago, IL) and GraphPad Prism version 5.0 software (GraphPad Software, LaJolla, CA). Group differences were determined using a two-tailed *t*-test for comparing two groups and a one-way Analysis of Variance (ANOVA) for comparing three or more groups. The sensitivity and specificity of *IGSF6* in diagnosing LUAD were analyzed using a receiver operating characteristic (ROC) curve, and the area under the curve (AUC) was estimated. Survival curves were analyzed using the log-rank test to calculate HR and *p*-value. The association of *IGSF6* expression with immune infiltrates was assessed using the Pearson correlation coefficient. The correlation between *IGSF6* expression and other molecules was analyzed using the Pearson correlation coefficient. The strength of correlation was categorized as very weak (r < 0.2), weak (r < 0.4), moderate (r < 0.6), strong (r < 0.8), or very strong (r < 1.0). Statistical significance was defined as *p* < 0.05.

## Results

### IGSF6 expression is decreased in LUAD

**Fig. 1:**
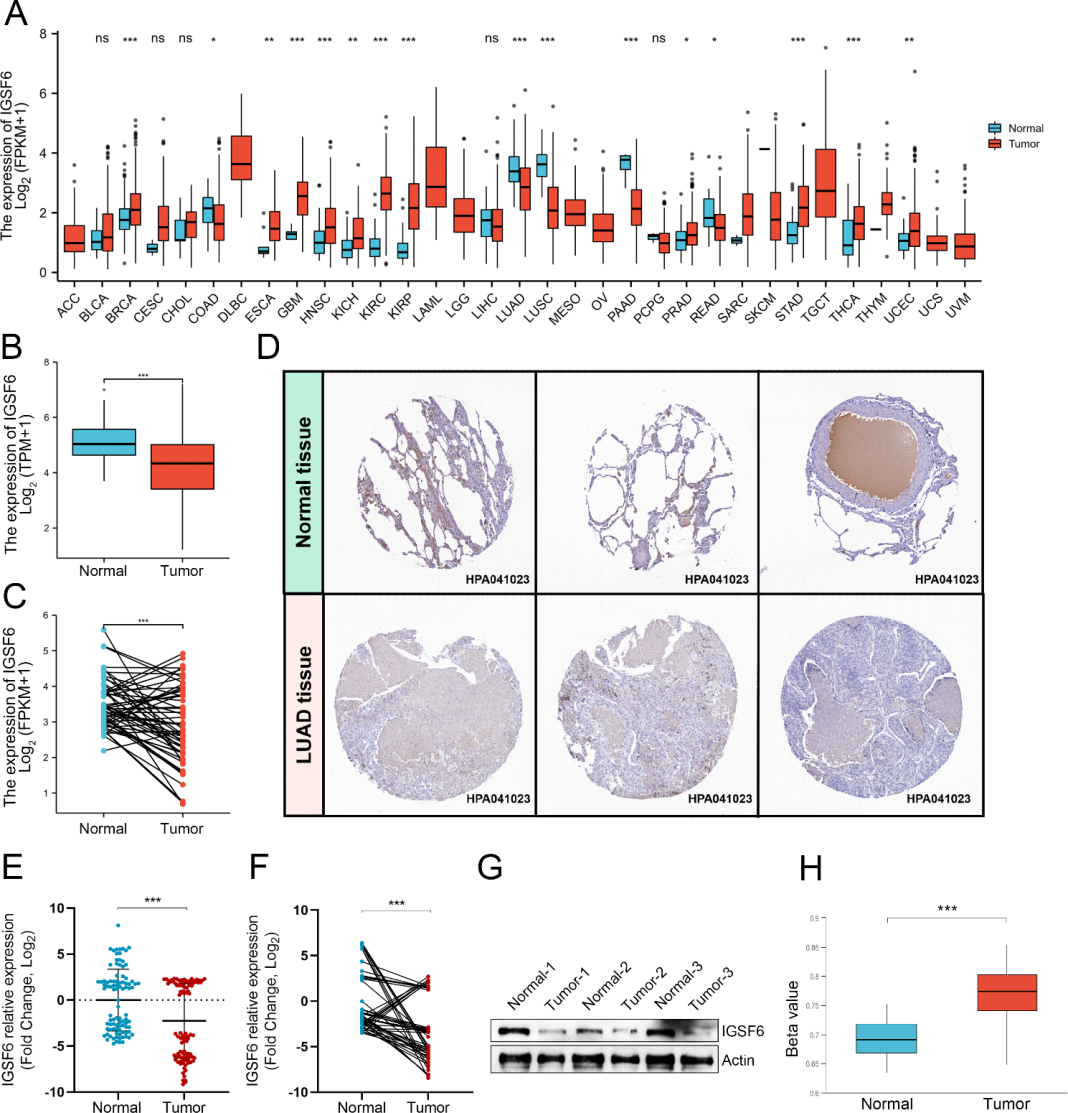
IGSF6 levels are decreased in LUAD. **(A)** The expression of *IGSF6* in different cancer types was determined using TIMER2.0 based on data from TCGA. **(B)** GEPIA2 was used to analyze the expression of *IGSF6* in LUAD tissues and normal tissues based on the data from TCGA. **(C)** The expression of *IGSF6* was examined in paired LUAD tissues and normal tissues based on the data from TCGA. **(D)** IHC images of IGSF6 protein in normal tissues and LUAD tissues were obtained from the HPA. **(E)***IGSF6* expression in tumor tissues and normal adjacent tissues that were collected from LUAD patients (n = 93). **(F)***IGSF6* expression in collected tumor tissues and paired normal adjacent tissues (n = 55). **(G)** IGSF6 protein levels in collected LUAD tissues and paired adjacent normal tissues (n = 3). **(H)** The methylation levels on the promoter of *IGSF6* in LUAD. Data were shown as mean ± SD. *p < 0.05, **p < 0.01, ***p < 0.001, ns: no significance

RNA-seq data from TCGA were used to analyze the *IGSF6* expression across 33 types of cancers. According to the analysis using TIMER2.0, *IGSF6* was indicated to be differently expressed in distinct tumors. It was suggested that *IGSF6* expression was increased in cancers such as bladder urothelial carcinoma (BLCA), esophageal carcinoma (ESCA), and glioblastoma multiforme (GBM), and was decreased in some others, particularly LUAD (Fig. 1A). To confirm the finding in LUAD, GEPIA2 was employed to analyze the expression of *IGSF6* in LUAD samples. The results demonstrated a significant decrease in *IGSF6* expression in LUAD samples (n = 535) compared to normal samples (n = 59) (Fig. 1B) (*p <* 0.001). Furthermore, when comparing LUAD tissues (n = 57) with their paired normal tissues (n = 57), a reduction in *IGSF6* expression was also observed (Fig. 1C) (*p* < 0.001). IHC images obtained from the HPA further supported these findings, showing minimal or undetectable levels of IGSF6 protein in LUAD tissues (Fig. 1D).

To validate the results obtained from the bioinformatics analysis, we conducted a study in which we measured the expression of *IGSF6* in tumor tissues (n = 93) and adjacent non-tumor tissues (n = 93) collected from LUAD patients. It was found that compared to that in adjacent non-tumor tissues, *IGSF6* expression was significantly decreased in LUAD tumor tissues (Fig. 1E) (*p* < 0.001). By further comparing the expression of *IGSF6* in paired tumor tissues and adjacent non-tumor tissues (n = 55), we found a significant downregulation of *IGSF6* expression in tumor tissues (Fig. 1F) (*p* < 0.001). In addition, we confirmed the decreased IGSF6 protein levels in tumor tissues compared to those in paired non-tumor tissues using western blotting (Fig. 1G).

To investigate the potential mechanism leading to the decreased expression of *IGSF6* in LUAD, we examined the methylation levels of the *IGSF6* promoter in normal tissues and primary tumors using UALCAN. Our analysis revealed a significant increase in methylation levels on the *IGSF6* promoter in primary LUAD tissues compared to normal tissues (Fig. 1H) (*p* < 0.001). This suggests that the elevated promoter methylation levels may contribute to the reduced expression of *IGSF6* in LUAD.

### IGSF6 is associated with the LUAD clinicopathological parameters

The association between *IGSF6* expression and clinical-pathological parameters in LUAD was initially analyzed by UALCAN, utilizing data from the TCGA dataset. The analysis revealed a negative correlation between *IGSF6* expression and TNM stages, as well as pathologic stages in LUAD (Additional file 3). We also analyzed the correlation between *IGSF6* expression and clinical-pathological parameters in LUAD based on data obtained from collected samples (n = 93). The results showed a consistent trend, where the expression of *IGSF6* decreased with tumor progression. Specifically, *IGSF6* expression was found to be negatively correlated with TNM stages and pathologic stages in LUAD (Table 1).

### IGSF6 is a potential biomarker for the diagnosis and prognosis of LUAD

**Fig. 2:**
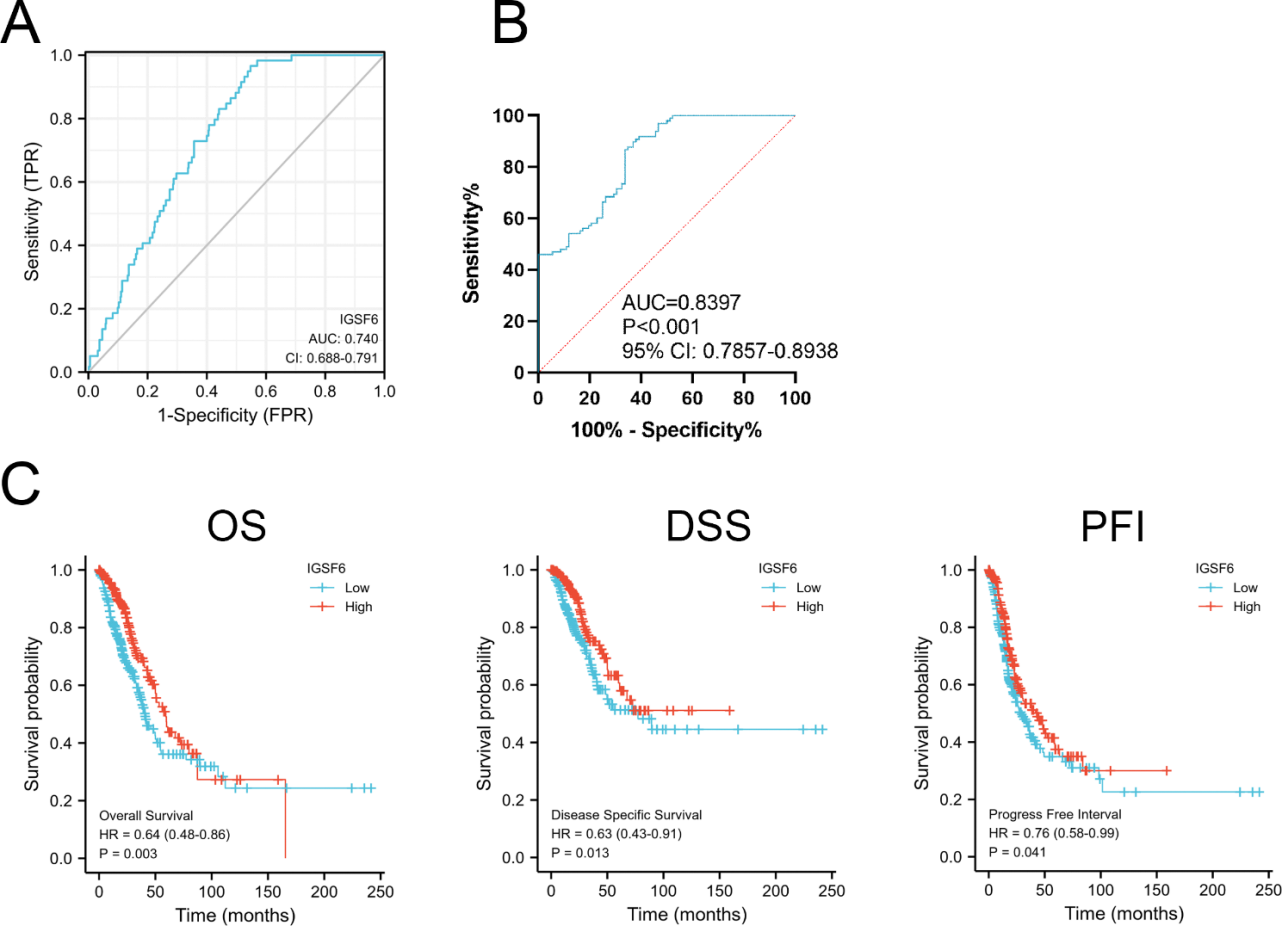
IGSF6 is a potential biomarker for the diagnosis and prognosis of LUAD. **(A, B)** ROC curve analysis of *IGSF6* in LUAD patients. **(C)** Kaplan–Meier survival curves were generated to compare the high-*IGSF6* and low-*IGSF6* cohorts in LUAD.

To identify the potential of IGSF6 in the diagnosis of LUAD, we obtained the *IGSF6* expressing profile in LUAD from TCGA and evaluated its diagnostic potential using the ROC curve. The AUC of ROC curve was 0.740 (95% CI 0.688–0.791) for *IGSF6* in LUAD diagnosis (Fig. 2A), indicating its potential as a diagnostic biomarker. We also assessed the diagnostic utility of *IGSF6* using data from our collected samples, and the AUC of the ROC curve was 0.8397 (95% CI 0.7857–0.8938) (Fig. 2B), further supporting its high sensitivity and specificity for LUAD diagnosis.

To evaluate the prognostic value of IGSF6 in LUAD, we analyzed the correlation between *IGSF6* expression and survival rates in the TCGA cohort. The results demonstrated that high *IGSF6* expression was significantly associated with favorable OS (HR = 0.64, *p* = 0.003), DSS (HR = 0.63, *p* = 0.013), and PFI (HR = 0.76, *p* = 0.041) (Fig. 2C). These findings indicate that IGSF6 is positively correlated with the improved prognosis of LUAD, suggesting that it may serve as a valuable prognostic biomarker for LUAD.

### IGSF6 is involved in the immune signature

Previous studies have suggested that IGSF6 is primarily expressed in immune system tissues and may function as a co-receptor in the antigen uptake or homing of DCs [19]. To investigate the potential role of IGSF6 in the anti-tumor immune response within LUAD, we performed enrichment analysis on the top 300 genes associated with *IGSF6* in LUAD.

**Fig. 3:**
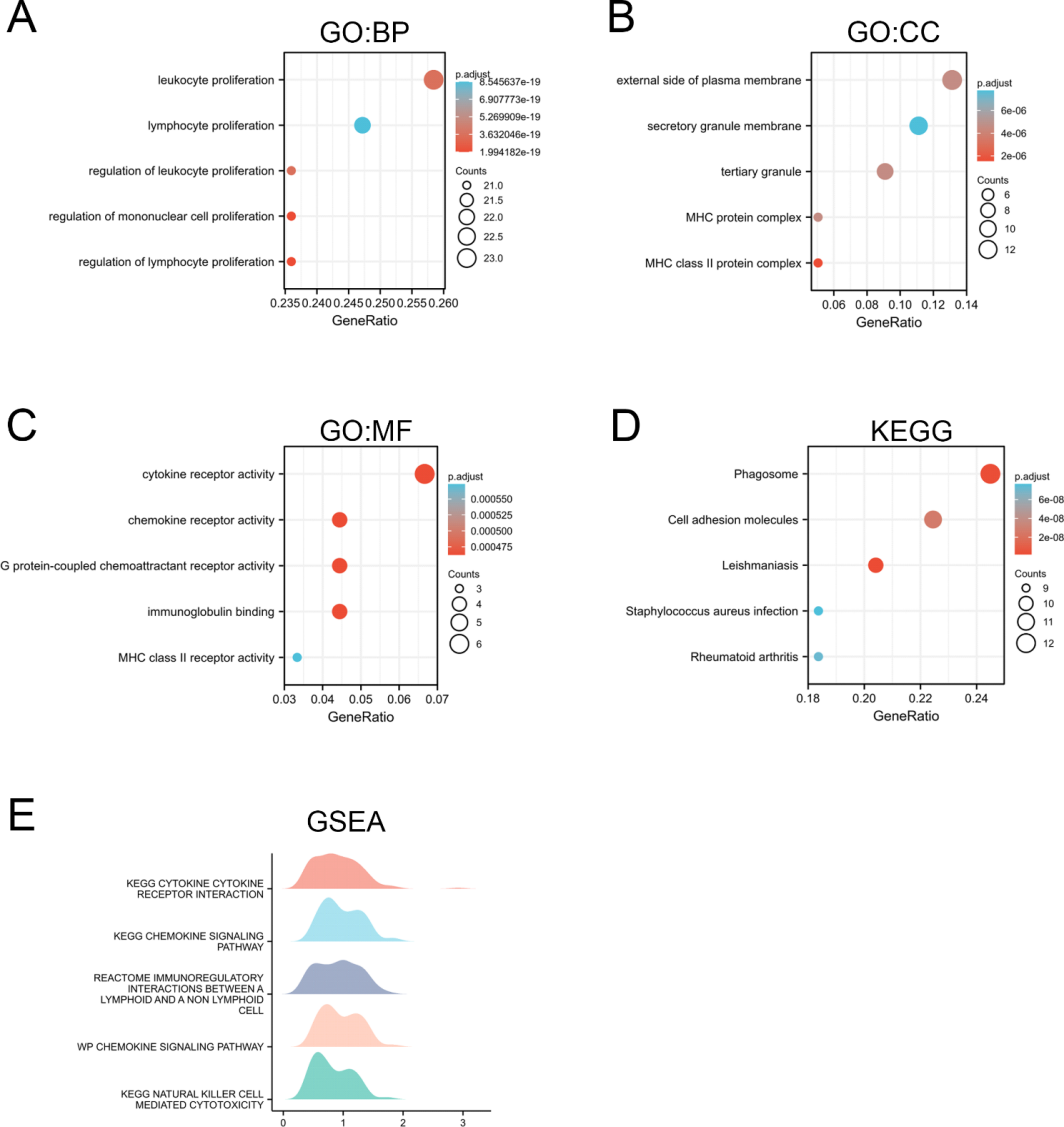
IGSF6 is involved in the immune signature. The top 300 genes associated with *IGSF6* in LUAD were selected for the enrichment analysis. **(A-C)** GO functional enrichment analyses including BP, CC, and MF were conducted. **(D)** KEGG pathways. **(E)** GSEA results

GO functional enrichment analysis was conducted using the R software *clusterProfiler* package. The analysis of biological processes (BP) indicated that IGSF6 was most closely associated with the proliferation of mononuclear cells, particularly lymphocytes (Fig. 3A). This suggests that IGSF6 may be involved in promoting the proliferation of immune cells in the TME. Cell component (CC) analysis showed that IGSF6 was predominantly located on the plasma membrane and was co-localized with major histocompatibility complex (MHC), which is a crucial marker of activated DCs and macrophages (Fig. 3B). This finding suggests that IGSF6 may be a cell membrane protein on DCs or macrophages, potentially playing a role in antigen presentation and immune response. Molecular function (MF) analysis identified that IGSF6 most likely functions as a receptor (Fig. 3C). This further supports the notion that IGSF6 may act as a receptor on immune cells, facilitating their interactions and signaling.

Further KEGG analysis suggested that IGSF6 was closely related to the formation of phagosome and cell adhesion, which are essential for antigen uptake and presentation by DCs and macrophages (Fig. 3D). This finding strengthens the hypothesis that IGSF6 may be involved in antigen processing and presentation. Additionally, GSEA analysis showed that gene sets related to cytokine-receptor interaction were significantly enriched in the high-*IGSF6* cohort (Fig. 3E). This suggests that IGSF6 may be involved in cytokine signaling pathways that regulate the function of immune cells.

Taken together, these analyses suggest that IGSF6 may function as a receptor on DCs or macrophages, playing a role in antigen processing and lymphocyte proliferation in the context of LUAD. These findings provide insights into the potential mechanisms underlying the involvement of IGSF6 in the anti-tumor immune response.

### IGSF6 is closely related to immune infiltrates with anti-tumor activity in LUAD

**Fig. 4:**
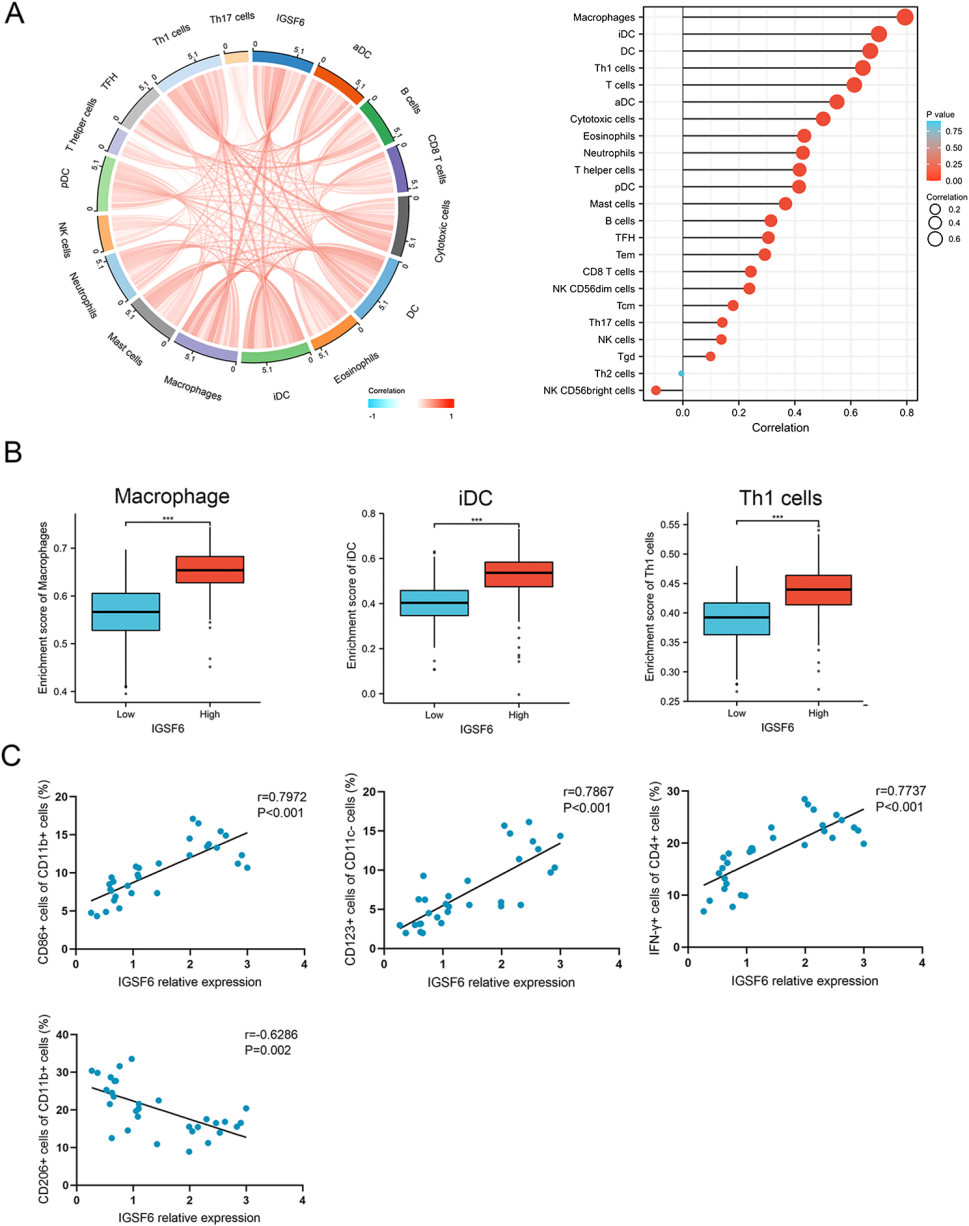
IGSF6 is associated with immune infiltrates in LUAD. **(A)** The correlation between *IGSF6* expression and the immune infiltrates in LUAD. **(B)** Infiltration scores of macrophages, iDCs, and Th1 in high- and low-*IGSF6* expression groups within LUAD. **(C)** The association of *IGSF6* expression with the percentage of M1, M2, iDCs, and Th1 in collected LUAD tissues (n = 33). Data were shown as mean ± SD. ***p < 0.001

To confirm the relationship between *IGSF6* expression and immune response in LUAD, TISIDB and TIMER2.0 were utilized to analyze the association of immune cell infiltration with *IGSF6* expression. As shown in Fig. 4A, macrophages, iDCs, and Th1 cells had the highest infiltration scores, which is consistent with previous studies indicating that IGSF6 expression is restricted to iDCs and myeloid cells. Furthermore, compared to the low-*IGSF6* cohort, the high-*IGSF6* cohort had significantly higher infiltration scores of macrophages (*p* < 0.001), iDCs (*p* < 0.001), and Th1 cells (*p* < 0.001) (Fig. 4B). We also found that *IGSF6* expression was positively correlated with the infiltration of M1 macrophages (r = 0.607, *p* < 0.001), but negatively correlated with the infiltration of M2 macrophages (r=-0.397, *p* < 0.001) in LUAD (Additional file 4).

To validate these findings, we detected the proportion of macrophages, iDCs, and Th1 cells in LUAD tissues (n = 33) and analyzed their correlation with *IGSF6* expression. The results showed that *IGSF6* expression was positively associated with the infiltration of M1 (r = 0.7972, *p* < 0.001), iDCs (r = 0.7876, *p* < 0.001), and Th1 cells (r = 0.7737, *p* < 0.001), while it was negatively associated with the percentage of M2 (r=-0.6286, *p* = 0.002) (Fig. 4C). These findings suggest that IGSF6 may play a critical role in promoting the anti-tumor activity in the TME of LUAD.

### IGSF6 is involved in the anti-tumor activity of M1 macrophages in LUAD

Based on the results in Fig. 3, it was suggested that IGSF6 might function as a receptor on iDCs or macrophages to regulate antigen processing and lymphocyte proliferation in LUAD. Additionally, it was revealed that *IGSF6* expression was positively related to the infiltration of M1 and iDCs in LUAD (Fig. 4). During the initiation of anti-tumor immune response, DCs and M1 macrophages play a crucial role in taking up and presenting tumor-associated antigens to Th1 cells, thereby activating T cell-induced immune responses [29, 30]. Based on these findings, we hypothesized that IGSF6 might affect the T cell-induced anti-tumor response by enhancing the activation and antigen processing of DCs and M1 in LUAD.

**Fig. 5:**
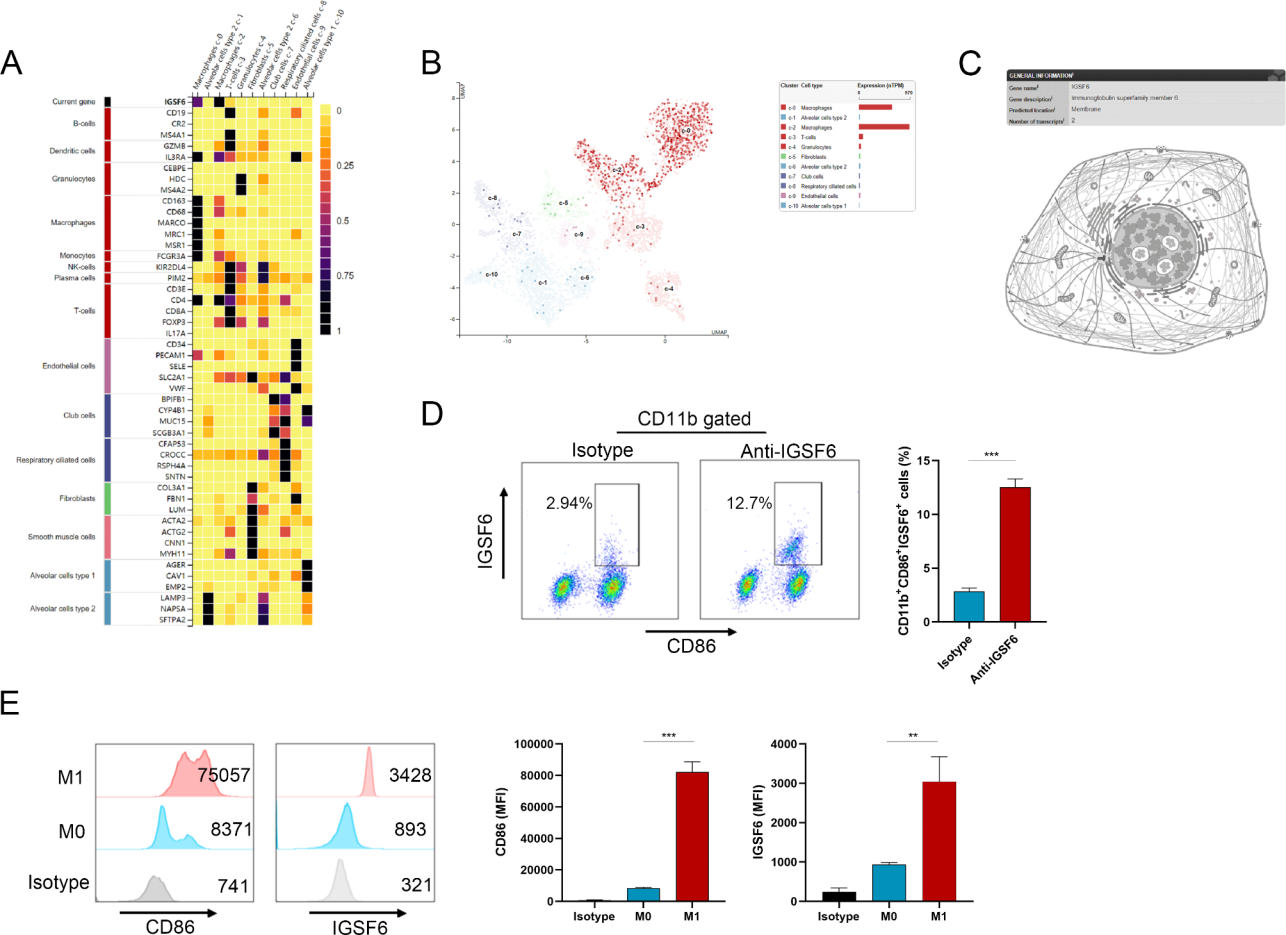
IGSF6 is localized on the membrane of M1 macrophages in LUAD. **(A)** Heatmap representation of the correlation between *IGSF6* expression and different cell types in lung tissues. **(B)** Analysis of *IGSF6* levels in different cell type groups derived from lung tissues. **(C)** Prediction of IGSF6 subcellular localization. **(D)** The IGSF6 levels on the plasma membrane of M1 macrophages from LUAD tissues. **(E)** The IGSF6 levels on the plasma membrane of induced M1 macrophages. Data were shown as mean ± SD. **p < 0.01, ***p < 0.001

To validate this hypothesis, we first identified the main cell population expressing IGSF6 in LUAD, which could be either macrophages or DCs. Using the HPA database, we analyzed the levels of IGSF6 in different cell types within lung tissues and found that IGSF6 was the most abundant in the cell population of macrophages (Fig. 5A-B). Subcellular localization analysis further indicated that IGSF6 was predominantly located on the plasma membrane of macrophages (Fig. 5C). Based on the results in Fig. 4, *IGSF6* expression was positively associated with the proportion of M1 macrophages, while negatively correlated with the frequency of M2 macrophages. Moreover, *IGSF6* expression was positively correlated with the expression of M1 markers and effectors (Additional file 5). Therefore, we detected the IGSF6 levels on M1 macrophages derived from LUAD tissues and confirmed the presence of IGSF6 protein (Fig. 5D) (*p* < 0.001). Furthermore, we induced M1 macrophages from THP-1 cells in vitro and observed an increase in the level of IGSF6 protein on the plasma membrane (*p* = 0.002), which corresponded to the polarization of M1 macrophages (Fig. 5E).

**Fig. 6:**
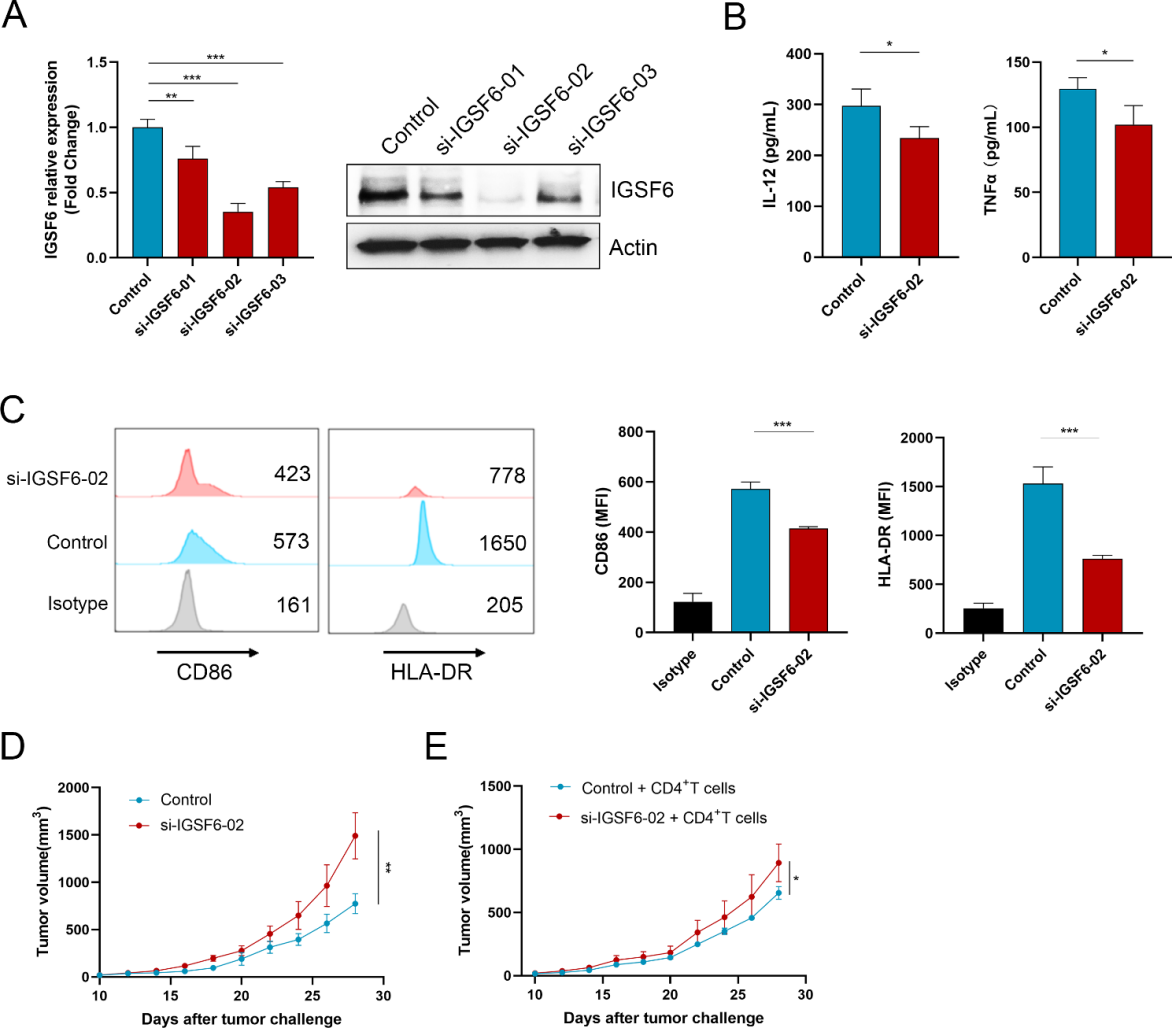
IGSF6 is involved in the anti-tumor activity of M1 macrophages in LUAD. **(A)** IGSF6 mRNA and protein levels in M1 macrophages with IGSF6 knockdown. **(B)** The production of IL-12 and TNFα by M1 macrophages with IGSF6 knockdown. **(C)** The levels of CD86 and HLA-DR on M1 macrophages with IGSF6 knockdown. **(D)** LUAD cells and M1 macrophages with IGSF6 knockdown were subcutaneously injected into nude mice (n = 6), and tumor progression was monitored over time. **(E)** CD4^+^ T cells, LUAD cells, and M1 macrophages with IGSF6 knockdown were injected into nude mice (n = 6), and tumor progression was measured at the indicated times. Data were shown as mean ± SD. *p < 0.05, **p < 0.01, ***p < 0.001

To investigate the impact of IGSF6 on M1 polarization and anti-tumor activity, we transfected M0 macrophages with specific siRNAs targeting IGSF6 and then observed the changes in M1 polarization and activity. The results demonstrated that the siRNAs effectively suppressed the expression of IGSF6, leading to a significant reduction in the production of TNF-α (*p* < 0.001) and IL-12 (*p* < 0.001) by M1 macrophages (Fig. 6A-B). Moreover, the knockdown of IGSF6 resulted in decreased levels of CD86 (*p* < 0.001) and HLA-DR (*p* < 0.001), which are markers for M1 macrophages (Fig. 6C). These findings suggest that IGSF6 plays a role in M1 polarization and anti-tumor activity.

To further investigate the impact of IGSF6 on the anti-tumor activity of M1 macrophages in vivo, we conducted experiments using nude mice. LUAD cells and M1 macrophages with IGSF6 knockdown were subcutaneously injected into the mice, and tumor progression was monitored over time. Compared to the control group, the si-IGSF6 group exhibited accelerated tumor progression (*p* = 0.0017) (Fig. 6D). Additionally, to confirm the role of IGSF6 in antigen processing by M1 macrophages in vivo, we injected CD4^+^ T cells, LUAD cells, and M1 macrophages with IGSF6 knockdown into nude mice and monitored tumor progression. The results showed that the si-IGSF6 group had enhanced tumor progression compared to the control group (*p* = 0.0229) (Fig. 6E). These findings provide further evidence of the regulatory role of IGSF6 in the anti-tumor activity of M1 macrophages.

## Discussion

IGSF6 is a novel member of the immunoglobulin superfamily, which belongs to the CD8 family of receptors and perhaps functions as a co-receptor [19]. The IGSF6 gene has been identified as a candidate for susceptibility to various diseases [31, 32]. However, its role in immune engagement during LUAD is not well understood. In this study, we identified that IGSF6 expression was significantly decreased in LUAD and was closely associated with clinicopathological parameters of the disease. We also identified IGSF6 as a potential biomarker for the diagnosis and prognosis of LUAD.

Previous research has characterized IGSF6 as a receptor for antigen uptake on iDCs. Mature DCs (mDCs), which efficiently present antigens to T cells, exhibit inhibited expression of *IGSF6* upon CD40L engagement [20]. In our study, we observed a positive correlation between *IGSF6* expression and the infiltration of iDCs in LUAD. However, we also found that *IGSF6* expression was positively correlated with the infiltration of activated Th1 cells that express high levels of CD40L in LUAD. This suggests that DCs may not be the primary cells expressing IGSF6 in LUAD. Based on data obtained from the HPA database, we predicted that IGSF6 is most abundant in macrophages within lung tissues. Furthermore, we confirmed the localization of IGSF6 on the plasma membrane of M1 macrophages in LUAD. This finding suggests that macrophages, particularly M1 macrophages, may be the primary cell type expressing IGSF6 in LUAD.

To further investigate the impact of IGSF6 on the anti-tumor activity of M1 macrophages, we used siRNA to knock down IGSF6 expression in M1 and then observed the change in cell biology. We found that IGSF6 knockdown significantly reduced the expression of CD86 and HLA-DR, suggesting that IGSF6 is responsible for antigen processing by M1 macrophages. Additionally, the knockdown of IGSF6 in M1 macrophages decreased the production of IL-12 and TNFα by M1 macrophages and accelerated the LUAD progression, indicating that IGSF6 is involved in the anti-tumor activity of M1 macrophages.

Previous research has shown that the expression of *IGSF6* in iDCs is inhibited by CD40L expressed by activated T cells [19]. There is also known intercellular interaction between macrophages and activated T cells expressing CD40L [33–35]. Interestingly, we found that IGSF6 levels increased along with the polarization of M1 macrophages, and our co-culture experiments with M1 macrophages and activated T cells did not show the CD40L-induced downregulation of *IGSF6* expression (Additional file 6). Moreover, as a type I membrane protein with a single Ig V_1_-J type loop in the extracellular domain, IGSF6 may interact principally with other adhesion or recognition molecules. These findings suggest that *IGSF6* expression in M1 macrophages may be mainly regulated by molecules produced by macrophages themselves. However, further investigation is needed to identify the specific molecules and related signaling pathways involved in this regulation.

## Conclusions

Our study has identified that IGSF6 could potentially serve as a biomarker for diagnosing and predicting the prognosis of LUAD. Furthermore, we have found that IGSF6 plays a critical role in the anti-tumor activity of M1 macrophages in LUAD.

### Electronic supplementary material

Below is the link to the electronic supplementary material.

Supplementary Material 1

Supplementary Material 2

Supplementary Material 3

Supplementary Material 4

Supplementary Material 5

Supplementary Material 6

## Data Availability

The datasets used and/or analyzed during the current study are available from the corresponding author on reasonable request.

## Abbreviations

IGSF6: Immunoglobulin superfamily 6
LUAD: Lung adenocarcinoma
TME: Tumor microenvironment
TNF-α: Tumor necrosis factor-alpha
IL-12: Interleukin-12
IL-6: Interleukin-6
IL-10: Interleukin-10
TGF-β: Transforming growth factor-beta
IGSF6: Immunoglobulin superfamily 6
DORA: DOwn-Regulated by Activation
DCs: Dendritic cells
TIMER2.0: Tumor immune estimation resource, version 2
GEPIA2: Gene expression profiling interactive analysis 2
IHC: Immunohistochemistry
OS: Overall survival
DSS: Disease special survival
PFI: Progress free interval
HR: Hazard ratio
GO: Gene Ontology
KEGG: Kyoto Encyclopedia of Genes and Genomes
GSEA: Gene set enrichment analysis
TISIDB: Tumor Immune System Interaction Database
FCM: Flow cytometry
ROC: Receiver operating characteristic curve
AUC: Area under the curve
TCGA: The Cancer Genome Atlas
ANOVA: Analysis of Variance
BLCA: Bladder urothelial carcinoma
ESCA: Esophageal carcinoma
GBM: Glioblastoma multiforme
BP: Biological processes
CC: Cell component
MHC: Major histocompatibility complex
MF: Molecular function
iDCs: Immature DCs
CD40L: CD40 ligand
mDCs: Mature DCs
