# Supplementary material for: Immunoglobulin superfamily 6 is a molecule involved in the anti-tumor activity of macrophages in lung adenocarcinoma
Source: BMC Cancer. 2023 Nov 30;23:1170. doi: 10.1186/s12885-023-11681-w (PMC10688083; doi:10.1186/s12885-023-11681-w)
Supplement: Supplementary file 1 — Supplementary Material 1 [file 12885_2023_11681_MOESM1_ESM.docx]

IGSF6 in tissue samples (Fig 1G):





β-ACTIN in tissue samples(Fig 1G)：





IGSF6 in cells with siRNA transfection(Fig 6A):





β-ACTIN in cells with siRNA transfection(Fig 6A):
