# Supplementary material for: Immunoglobulin superfamily 6 is a molecule involved in the anti-tumor activity of macrophages in lung adenocarcinoma
Source: BMC Cancer. 2023 Nov 30;23:1170. doi: 10.1186/s12885-023-11681-w (PMC10688083; doi:10.1186/s12885-023-11681-w)
Supplement: Supplementary file 2 — Supplementary Material 2 [file 12885_2023_11681_MOESM2_ESM.docx]

**Additional file 2**


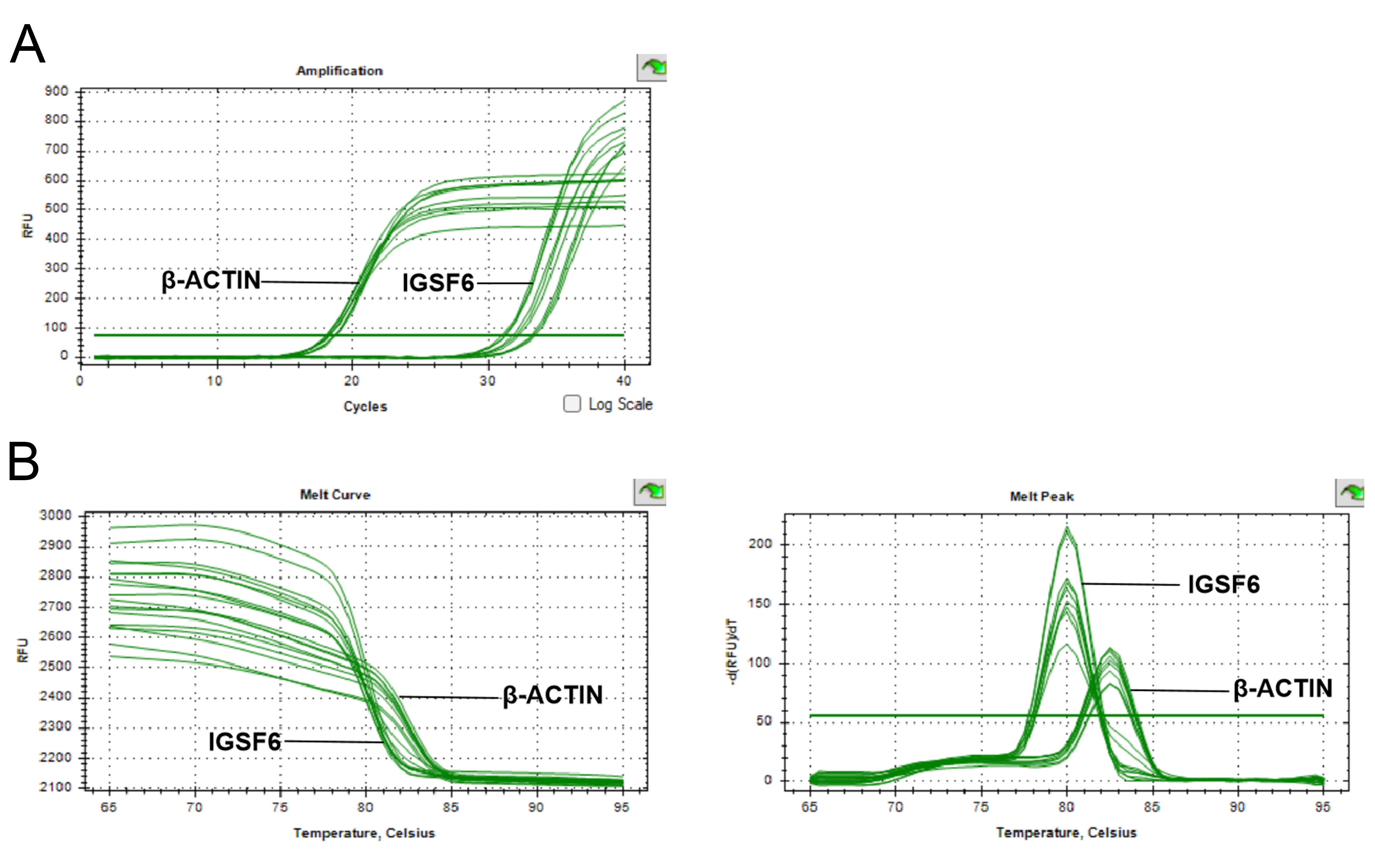


**Fig S1** Representative amplification plot and melt curve. Total RNA was isolated from LUAD tissues (n=3) and was reverse-transcribed into cDNA to perform qRT-PCR. Each sample had three replicates. (A) Amplification plot. (B) Melt Curve and Melt Peak.
