# Supplementary material for: Immunoglobulin superfamily 6 is a molecule involved in the anti-tumor activity of macrophages in lung adenocarcinoma
Source: BMC Cancer. 2023 Nov 30;23:1170. doi: 10.1186/s12885-023-11681-w (PMC10688083; doi:10.1186/s12885-023-11681-w)
Supplement: Supplementary file 3 — Supplementary Material 3 [file 12885_2023_11681_MOESM3_ESM.docx]

**Additional file 3**

Table S1. Correlation between *IGSF6* expression and clinicopathological characteristics of LUAD patients

| Characteristics | Total(N) | Univariate analysis | |  | Multivariate analysis | |
| --- | --- | --- | --- | --- | --- | --- |
|  | | Hazard ratio (95% CI) | *p*value |  | Hazard ratio (95% CI) | *p*value |
| **Age** | 516 |  |  |  |  |  |
| <=65 | 255 | Reference |  |  |  |  |
| >65 | 261 | 1.223 (0.916-1.635) | 0.172 |  |  |  |
|  |  |  |  |  |  |  |
| **Gender** | 526 |  |  |  |  |  |
| Female | 280 | Reference |  |  |  |  |
| Male | 246 | 1.070 (0.803-1.426) | 0.642 |  |  |  |
|  |  |  |  |  |  |  |
| **IGSF6** | 526 |  |  |  |  |  |
| Low | 262 | Reference |  |  |  |  |
| High | 264 | 0.642 (0.480-0.860) | **0.003** |  | 0.648 (0.458-0.917) | **0.014** |
|  |  |  |  |  |  |  |
| **T stage** | 523 |  |  |  |  |  |
| T1 | 175 | Reference |  |  |  |  |
| T2 | 282 | 3.326 (1.751-6.316) | **0.020** |  | 3.124 (1.533-6.365) | 0.079 |
| T3 | 47 | 2.937 (1.746-4.941) | **<0.001** |  | 2.045 (0.894-4.677) | **0.002** |
| T4 | 19 | 1.521 (1.068-2.166) | **<0.001** |  | 1.506 (0.954-2.378) | 0.090 |
|  |  |  |  |  |  |  |
| **M stage** | 377 |  |  |  |  |  |
| M0 | 352 | Reference |  |  |  |  |
| M1 | 25 | 2.136 (1.248-3.653) | **0.006** |  | 1.671 (0.753-3.707) | 0.207 |
|  |  |  |  |  |  |  |
| **N stage** | 510 |  |  |  |  |  |
| N0 | 343 | Reference |  |  |  |  |
| N1 | 80 | 3.108 (2.136-4.521) | **<0.001** |  | 2.395 (1.252-4.580) | **0.008** |
| N2 | 71 | 2.381 (1.695-3.346) | **<0.001** |  | 2.202 (0.938-5.170) | 0.070 |
| N3 | 16 | 1.617 (1.076-2.249) | **<0.001** |  | 1.586 (1.123-2.317) | **0.003** |
|  |  |  |  |  |  |  |
| **Pathologic stage** | 518 |  |  |  |  |  |
| Stage I | 290 | Reference |  |  |  |  |
| Stage II | 121 | 3.790 (2.193-6.548) | **<0.001** |  | 1.317 (0.505-3.434) | 0.572 |
| Stage III | 81 | 3.544 (2.437-5.154) | **<0.001** |  | 0.817 (0.406-1.646) | 0.574 |
| Stage IV | 26 | 2.418 (1.691-3.457) | **<0.001** |  |  |  |
