# Supplementary material for: Immunoglobulin superfamily 6 is a molecule involved in the anti-tumor activity of macrophages in lung adenocarcinoma
Source: BMC Cancer. 2023 Nov 30;23:1170. doi: 10.1186/s12885-023-11681-w (PMC10688083; doi:10.1186/s12885-023-11681-w)
Supplement: Supplementary file 4 — Supplementary Material 4 [file 12885_2023_11681_MOESM4_ESM.docx]

**Additional file 4**


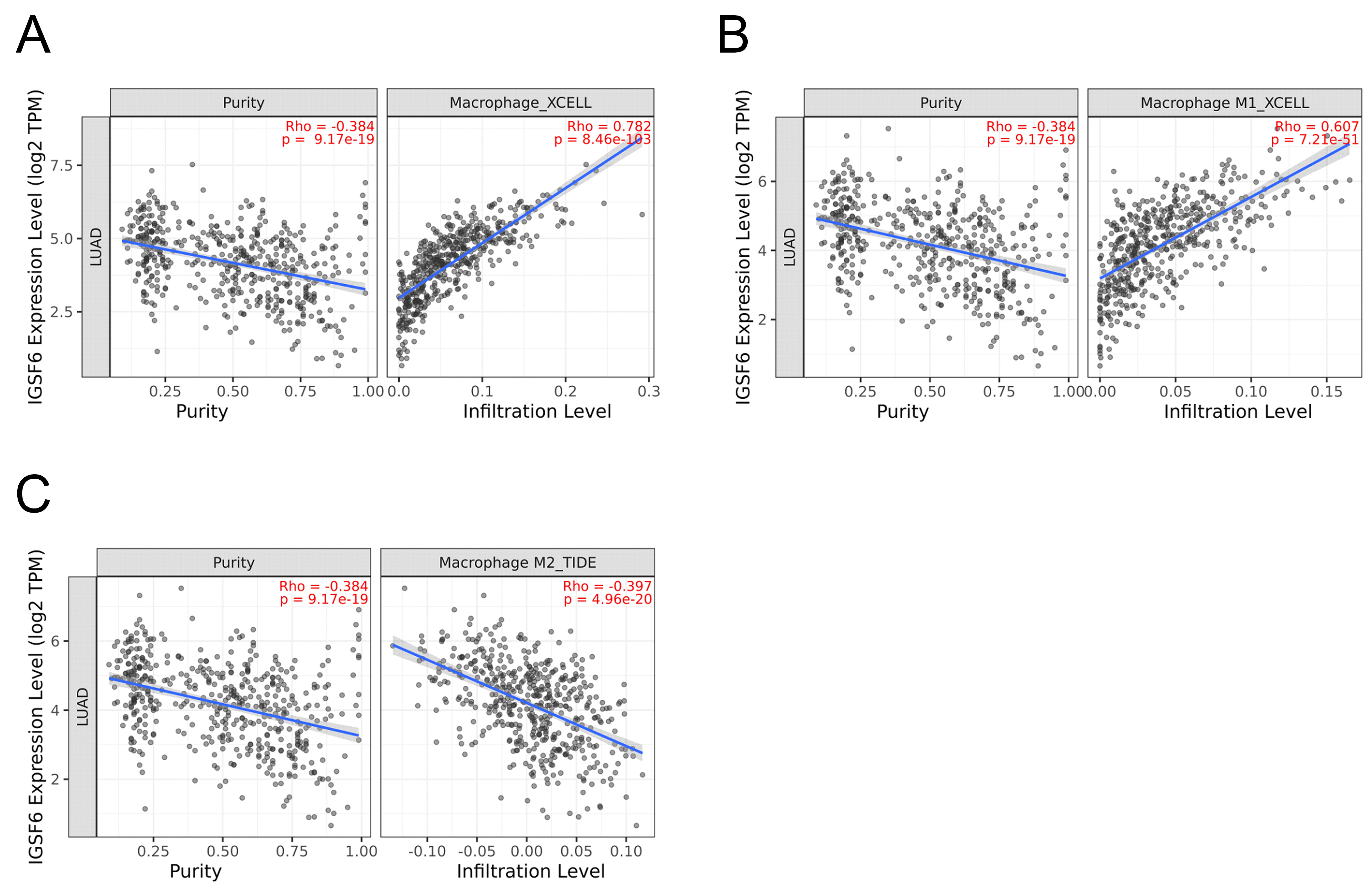


**Fig S2** Correlation between *IGSF6* expression and different subsets of macrophages. (A) Total macrophages. (B) M1 macrophages. (C) M2 macrophages.
