# Supplementary material for: Immunoglobulin superfamily 6 is a molecule involved in the anti-tumor activity of macrophages in lung adenocarcinoma
Source: BMC Cancer. 2023 Nov 30;23:1170. doi: 10.1186/s12885-023-11681-w (PMC10688083; doi:10.1186/s12885-023-11681-w)
Supplement: Supplementary file 5 — Supplementary Material 5 [file 12885_2023_11681_MOESM5_ESM.docx]

**Additional file 5**


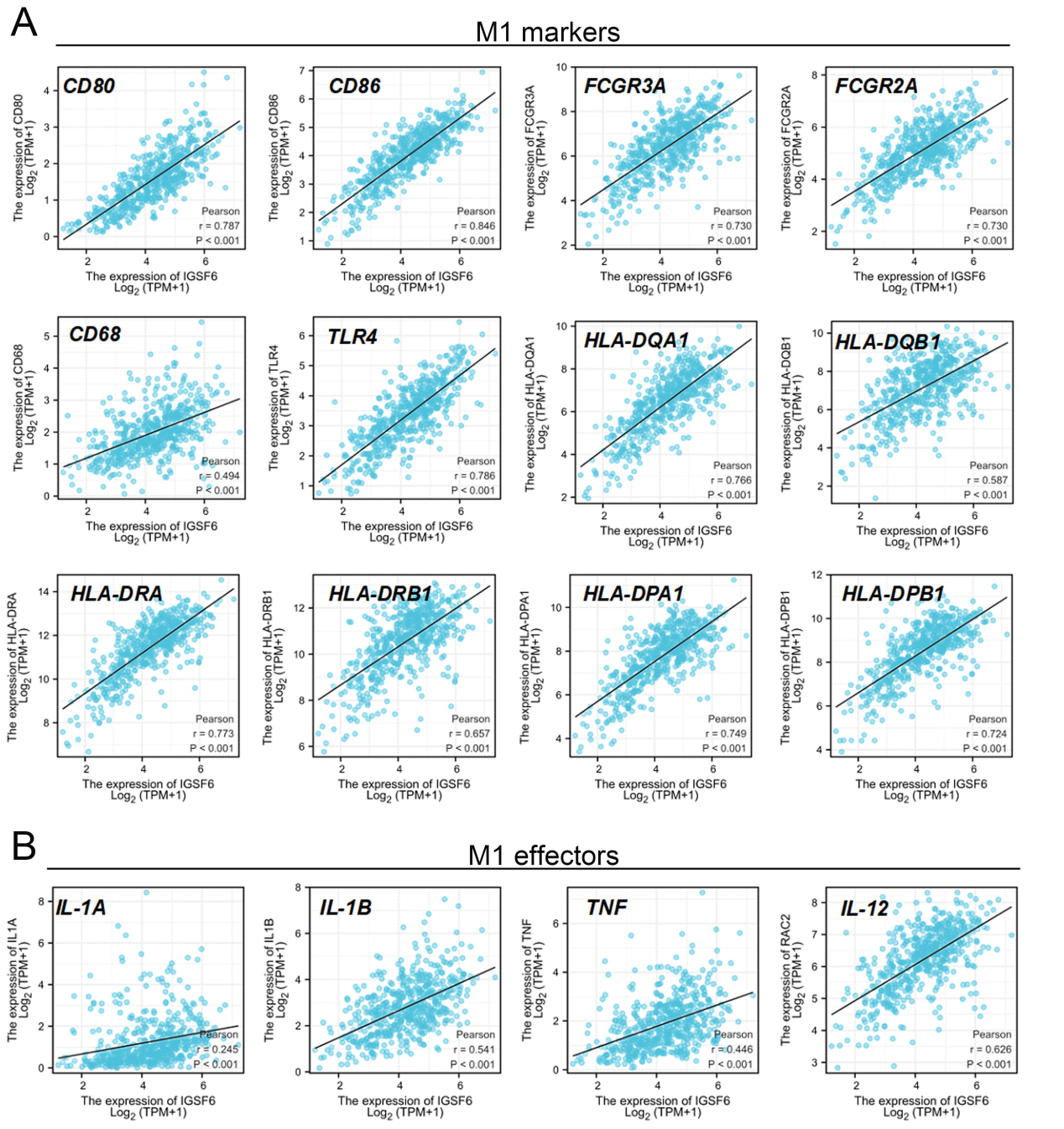


**Fig S3** *IGSF6* expression is associated with M1 macrophages. **(A)** Correlation between *IGSF6* expression and genes encoding M1 markers. **(B)** Correlation between *IGSF6* expression and genes encoding M1 effectors.
