# Supplementary material for: Immunoglobulin superfamily 6 is a molecule involved in the anti-tumor activity of macrophages in lung adenocarcinoma
Source: BMC Cancer. 2023 Nov 30;23:1170. doi: 10.1186/s12885-023-11681-w (PMC10688083; doi:10.1186/s12885-023-11681-w)
Supplement: Supplementary file 6 — Supplementary Material 6 [file 12885_2023_11681_MOESM6_ESM.docx]

**Additional file 6**


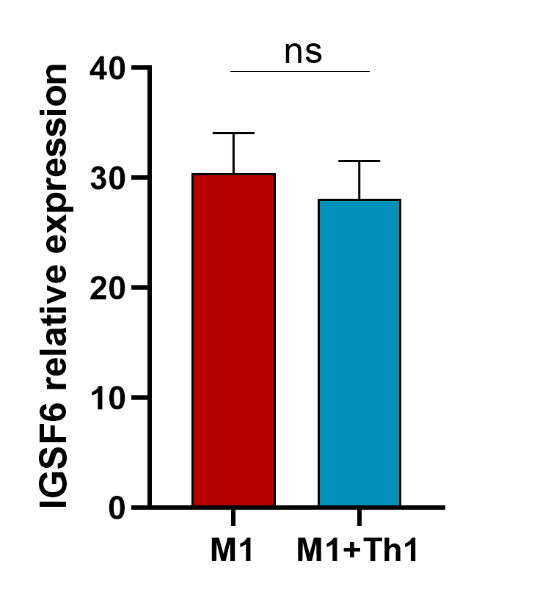


**Fig S4** *IGSF6* expression in M1 macrophages is not affected by activated Th1. Induced M1 were co-cultured with CD4^+^ T cells activated by CD3 + CD28, and then qRT-PCR was performed to detect the expression of *IGSF6*.
